# Late Transcatheter Aortic Valve Thrombosis Leading to Cardiogenic Shock

**DOI:** 10.1016/j.jaccas.2022.07.032

**Published:** 2022-11-16

**Authors:** Qasim Al Abri, Lamees I. El Nihum, Tomoya Hinohara, Su Min Chang, Nadeen N. Faza, Sachin S. Goel, Neal S. Kleiman, Moritz C. Wyler von Ballmoos, Marvin D. Atkins, Michael J. Reardon

**Affiliations:** a DeBakey Heart & Vascular Center, Houston Methodist Hospital, Houston, Texas, USA; b Texas A&M College of Medicine, Bryan, Texas, USA

**Keywords:** hypoattenuated leaflet thrombosis, transcatheter heart valve, CT, computed tomography, DAPT, dual antiplatelet therapy, HALT, hypoattenuated leaflet thrombosis, IABP, intra-aortic balloon pump, LVEF, left ventricular ejection fraction, OAC, oral anticoagulation, SAPT, single antiplatelet therapy, SAVR, surgical aortic valve replacement, TAV, transcatheter aortic valve, TAVR, transcatheter aortic valve replacement, TEE, transesophageal echocardiography, TTE, transthoracic echocardiography

## Abstract

A 67-year-old woman with prior transcatheter aortic valve replacement presented with worsening dyspnea. Imaging revealed transcatheter aortic valve thrombosis and aortic stenosis. Despite oral anticoagulation, she progressively deteriorated and developed cardiogenic shock. We highlight the Heart Team’s role in treating this unusual late thrombosis. (**Level of Difficulty: Intermediate.**)

## Central Illustration

**Figure:**
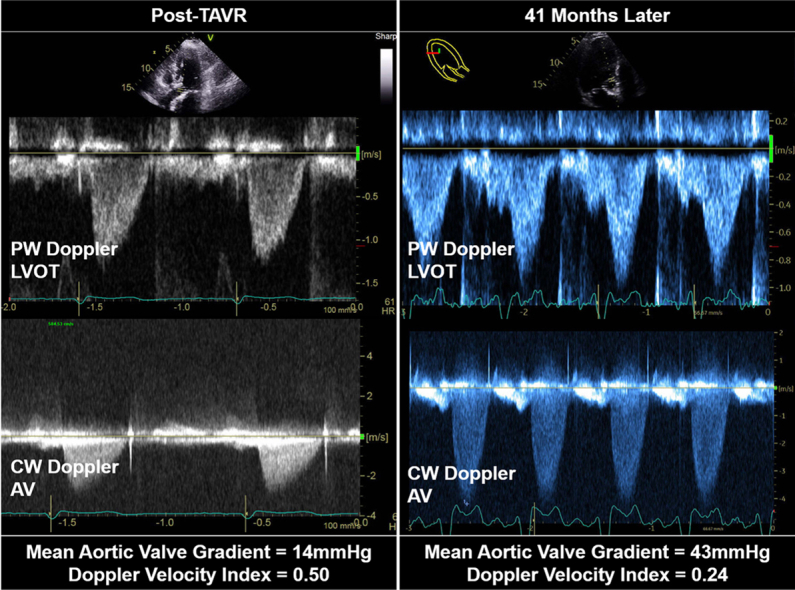

## History of Presentation

A 67-year-old woman presented to the emergency department with a 2-week history of progressive shortness of breath. On physical examination, temperature was 97.4 °F, heart rate was 74 beats/min, blood pressure was 92/57 mm Hg, and respirations were 20 per minute with oxygen saturation of 92% on room air. She had a 4/6 systolic murmur, and expiratory wheezes were heard bilaterally. Peripheral pulses were palpable, and there was no edema. She was not in acute distress.

**Learning Objectives**

• To illustrate a rare case of unusual late transcatheter aortic valve thrombosis presenting with cardiogenic shock.
• To highlight the role of transcatheter aortic valve explantation and surgical aortic valve replacement in patients for whom medical management of valve thrombosis is inadequate.
• To emphasize that further studies are needed to determine the optimal individualized antithrombotic regimen after transcatheter aortic valve replacement.

## Past Medical History

She underwent transcatheter aortic valve replacement (TAVR) with a 23-mm Evolut Pro (Medtronic) 41 months earlier and was maintained on dual antiplatelet therapy (DAPT). Despite her age at initial implantation, the patient had multiple comorbidities and a very complex medical history. This included severe peripheral vascular disease with aorto-bifemoral bypass that had occluded, requiring a redo aorto-bifemoral graft. She also had multiple strokes and bilateral carotid stents, poor pulmonary function tests with obstructive features due to prolonged history of smoking, late-stage chronic kidney disease, and chronic pain with a spinal stimulator. She was thoroughly evaluated by our heart team, and the decision was made to proceed with TAVR based on both perceived likelihood of a very difficult recovery from surgery as well as likely limited life expectancy of <10 years.

## Investigations

**Figure 1:**
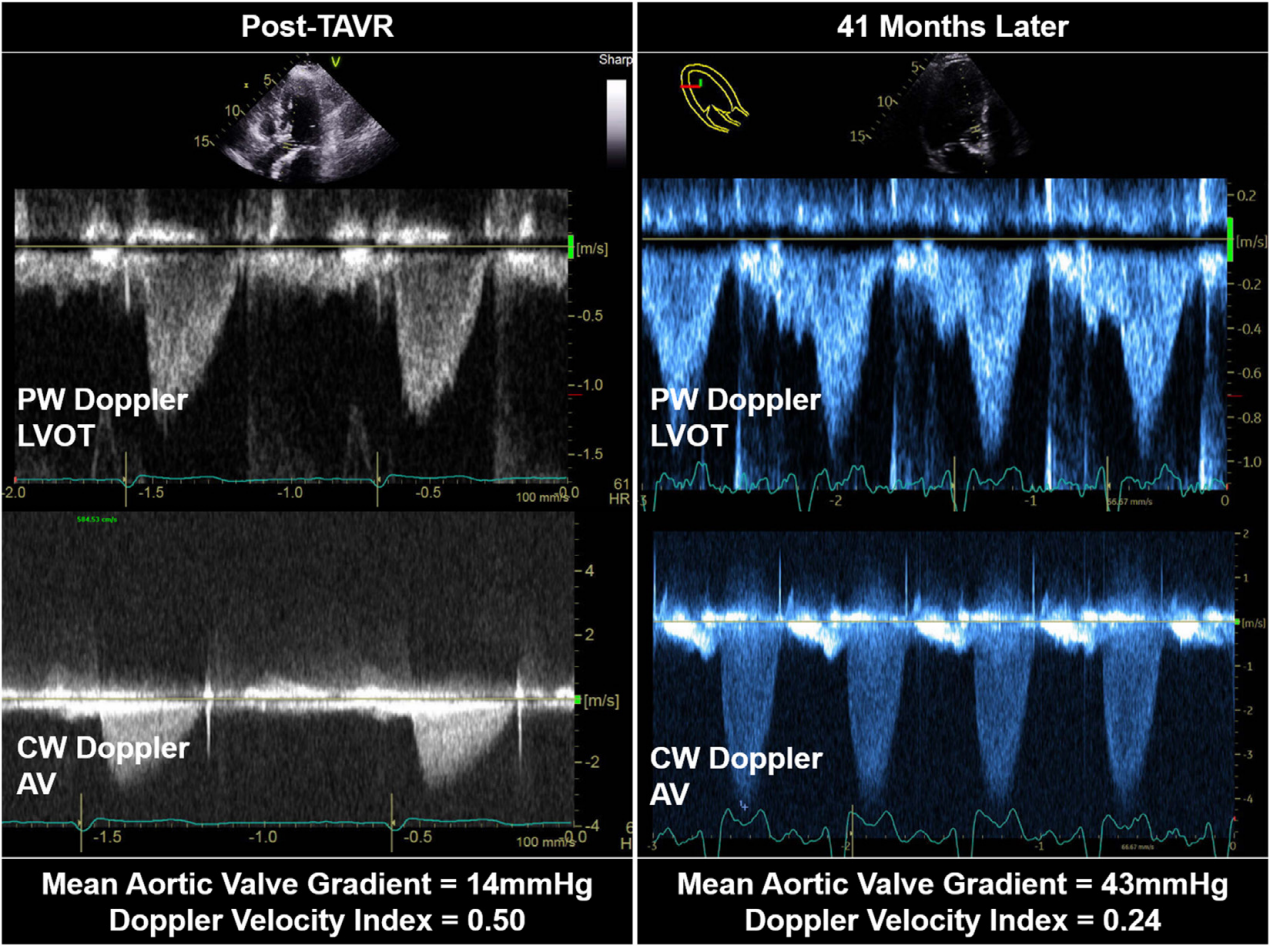
Transcatheter Aortic Valve Doppler Echocardiography Pulsed-wave (PW) Doppler echocardiography of the left ventricular outflow tract (LVOT) and continuous-wave (CW) Doppler echocardiography of the transcatheter aortic valve (AV) demonstrated progression of aortic stenosis 41 months after transcatheter aortic valve replacement (TAVR).

**Figure 2:**
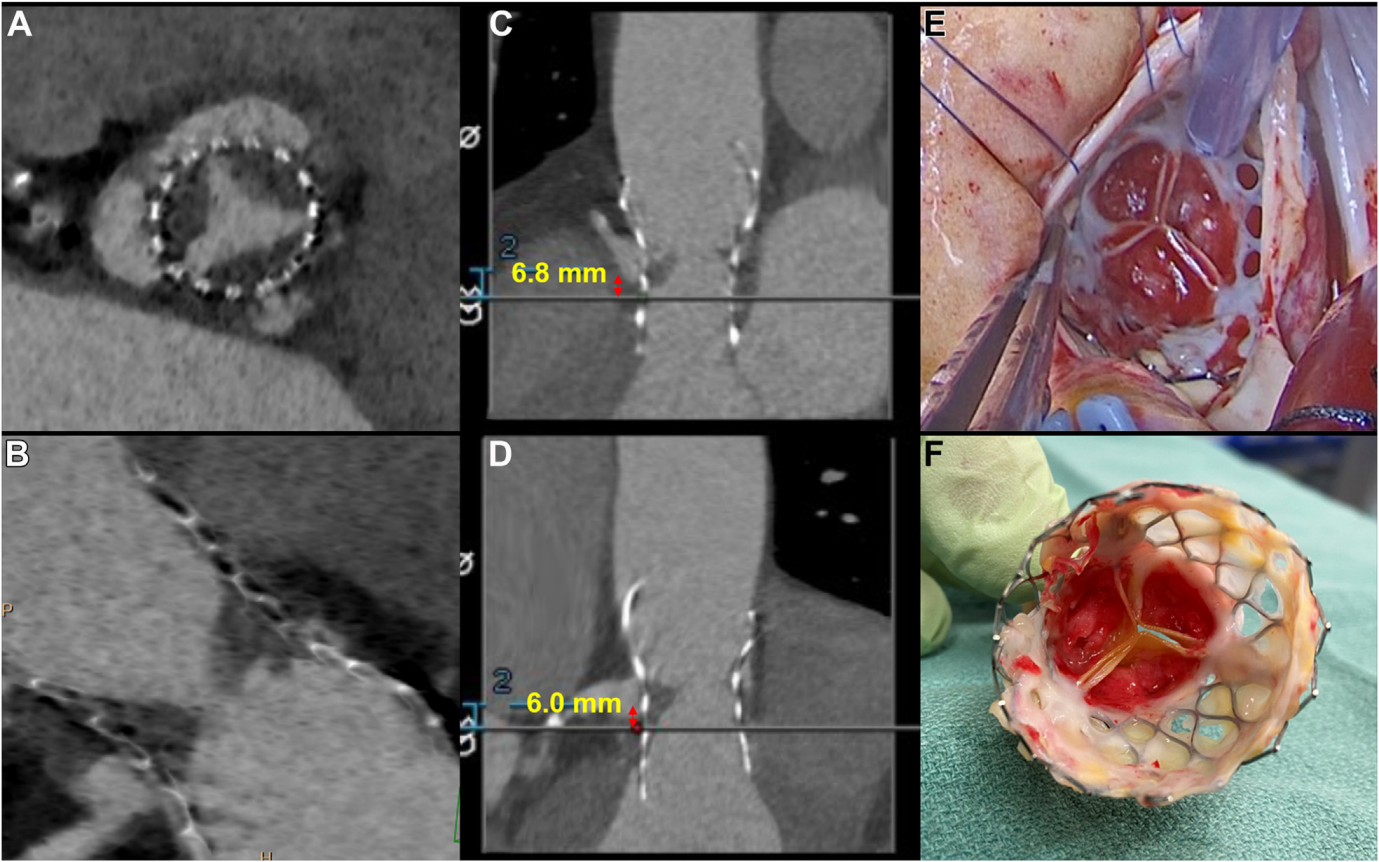
Transcatheter Aortic Valve Thrombosis **(A)** Short-axial and **(B)** long-axial computed tomography demonstrated 3-leaflet thrombosis of the transcatheter aortic valve and low coronary heights of the **(C)** right coronary artery and **(D)** left coronary artery. Intraoperative inspection of the transcatheter aortic valve showed thrombosis of all leaflets on both the inflow and the outflow **(E and F)**.

Transthoracic echocardiography (TTE) showed left ventricular ejection fraction (LVEF) of 30%-35%, peak aortic valve velocity of 4 m/s, mean gradient of 43 mm Hg, and dimensionless valve index (DVI) of 0.24, consistent with severe aortic stenosis. This was concerning for TAV thrombosis given previous TTE findings of normal LVEF, mean aortic gradient of 14 mm Hg, and DVI of 0.50 after TAVR (Figure 1, Video 1). Cardiac computed tomography (CT) confirmed 3-leaflet TAV thrombosis (Figures 2A and 2B, Video 2). The valve depth was 6 mm from the annulus.

## Management

She was started on oral anticoagulation (OAC) with warfarin with a plan for close outpatient follow-up with the heart team. After discharge, her international normalized ratio was never subtherapeutic and ranged between 3.0 to 4.0 with 1 episode of supratherapeutic levels of 6.0 to 7.0 without bleeding. However, she failed to improve and presented 2 weeks later with cardiogenic shock (SCAI shock stage C) requiring an intra-aortic balloon pump (IABP) and inotropic support. Her LVEF according to TTE had dropped to 20%-25%. She also had bowel ischemia and shock liver which were judged to be due to low cardiac output and managed conservatively. With inotropic and mechanical support, the patient stabilized.

The patient’s critical condition and the need for urgent intervention were reviewed by the heart team. Because of her prohibitive aortic root anatomy with respect to risk of coronary occlusion and risk of embolization, a TAV-in-TAV procedure was excluded (Figures 2C and 2D). We elected to proceed with surgical explantation of the TAV and surgical aortic valve replacement (SAVR), despite the high surgical risk and complexity.

Following cardiopulmonary bypass (CPB), arrest, and aortotomy, surgical explantation was started with careful and meticulous initiation of the plane circumferentially between the TAV and the aorta. Once the native aortic valve leaflets were reached, dissection was continued between the native leaflets and the TAV, extending the plane down into the left ventricular outflow tract. The TAV was fully explanted, the native aortic valve leaflets were excised, and SAVR was performed with the use of a 23-mm Inspiris pericardial valve (Edwards Lifesciences). The aortotomy was closed, and the patient was weaned from CPB. Transesophageal echocardiography (TEE) showed a mean aortic valve gradient of 5 mm Hg with no perivalvular leak.

Inspection of the TAV showed thrombosis of all leaflets both on the inflow and the outflow (Figures 2E and 2F).

She was extubated the same day, and her IABP was weaned and removed the next day in the operating room with repair of the aorto-bifemoral graft insertion site. She continued to make good recovery and was discharged home on postoperative day 12 on warfarin.

## Discussion

Symptomatic TAV thrombosis is rare, and can be associated with serious consequences such as stroke, congestive heart failure, cardiogenic shock, and death. Although leaflet thrombosis after TAVR occurs relatively frequently, the majority of patients follow a subclinical course, and both asymptomatic and symptomatic cases have demonstrated resolution of hypoattenuated leaflet thickening (HALT) after anticoagulation treatment.^1^^,^^2^ However, in many cases medical management with OAC is not sufficient, and a decision for transcatheter or surgical intervention should ideally occur before the patient presents with worsening symptoms, such as the presentation of cardiogenic shock in the present patient. We highlight the crucial role of the heart team in proceeding with TAV explantation and SAVR in the management of such patients.

The characteristic finding of HALT on postoperative TAVR-protocol CT is a hypoattenuating opacity at the base of valve leaflets, reported in up to 16% of patients 1 month after TAVR and 27.5% 1 year after TAVR.^3^ Studies continue to evaluate whether HALT is without clinical consequence or whether it should alter clinical management, although most studies indicate a lack of association between HALT and adverse clinical events.^3^ The question remains, however, whether HALT is an indicator of earlier-onset structural valve deterioration, thus warranting interventions to prevent or reverse it.^3^ Though standard treatment is anticoagulation, HALT has been shown to progress and regress over time with or without anticoagulation, and it is not clear that an anticoagulation strategy limited to a 3- to 6-month window after TAVR will meaningfully affect the incidence of HALT after that window has passed.^3^ Despite our patient’s excellent initial post-TAVR metrics and adherence to DAPT protocol for over 3 years, she surprisingly presented 41 months after TAVR with late thrombosis, severe aortic stenosis, and rapidly progressive symptoms.

The mechanism behind HALT following TAVR remains unclear, with theories for the increased prothrombotic activity following TAVR including coagulant activity of the remnant native valve; the intra-annular design of the prostheses causing dead-water areas favoring thrombosis; high shear stress at the metal struts potentially causing blood trauma and increased procoagulant activity; and an increased on-clopidogrel platelet activity in the elderly.^1^ In addition, anatomic factors such as valve sizing and deep implantation of the valve may contribute to HALT. The initial TAVR implant depth was deep at about 8 mm, but its relationship to valve thrombosis is not clear in this individual case. The patient’s history of occluded aorta in the past and possible underlying hypercoagulability may have contributed to her presentation.

In patients with HALT presenting with heart failure, various modes of medical management have been reported in the literature. Kefer et al^4^ reported successful treatment of HALT with heparin and warfarin in a patient who presented with heart failure and a TAV peak gradient of 73 mm Hg 4 months after TAVR. The patient had undergone DAPT for 1 month after TAVR before moving to single antiplatelet therapy (SAPT).^4^ Leetmaa et al^2^ described a patient on SAPT who presented with severe decompensated heart failure 44 days after TAVR. He was treated with unfractionated heparin infusion and low-molecular-weight heparin and warfarin but developed refractory heart failure and died on day 137 after TAVR.^2^ It is unclear whether TAV-in-TAV or TAV explantation and SAVR were considered in that patient. Finally, TAV explantation and SAVR have been reported in patients presenting with heart failure without overt cardiogenic shock.^5^

HALT is a potentially reversible cause of valve dysfunction and thromboembolism.^2^ Leetmaa et al^2^ emphasize the role of early diagnosis and treatment in the prevention of valve stenosis and thromboembolic events, and suggest follow-up CT as a valuable tool for leaflet thrombosis detection, supplementing both standard TTE and TEE. In addition, further studies are needed to determine the optimal individualized post-TAVR antithrombotic regimen.^2^ SAPT with aspirin, DAPT with aspirin and clopidogrel, and OAC are used as antithrombotic regimens following TAVR. The present case demonstrates the need for continued study into post-TAVR antithrombotic regimen. Recently, OAC was evaluated for post-TAVR anticoagulation and was shown to be more effective than an antiplatelet-based therapy in preventing reduced leaflet motion at 90 days.^6^ A recent meta-analysis by Bogyi et al^7^ highlighted the lower risk of subclinical leaflet thrombosis with indication-based use of OAC after TAVR compared with SAPT and DAPT.

## Follow-Up

At 4-month follow-up, the patient was doing well with resolution of her symptoms.

## Conclusions

Late symptomatic TAV thrombosis with cardiogenic shock is a rare complication of TAVR. Careful evaluation of patient anatomy and deliberation of the heart team over treatment options, including TAV explantation and SAVR by a skilled surgical team, are paramount in management when TAV-in-TAV is not feasible.

## Funding Support and Author Disclosures

Dr Goel is a consultant to Medtronic; and is on the Speakers Bureau for Abbott Structural Heart. Dr Wyler von Ballmoos is a consultant to Medtronic and Boston Scientific. Dr Reardon is a consultant to Medtronic, Boston Scientific, and Gore Medical. All other authors have reported that they have no relationships relevant to the contents of this paper to disclose.

## Appendix

Supplemental Video 1 Angiography of the Index Transcatheter Aortic Valve Replacement Procedure

Supplemental Video 2 Short-Axial and Long-Axial Computed Tomography Showing 3-Leaflet Thrombosis of the Transcatheter Aortic Valve
